# Rational Application of First-Line EGFR-TKIs Combined with Antiangiogenic Inhibitors in Advanced EGFR-Mutant Non-Small-Cell Lung Cancer: A Systematic Review and Meta-Analysis

**DOI:** 10.1155/2021/8850256

**Published:** 2021-01-28

**Authors:** Jie-Tao Ma, Yi-Jia Guo, Jun Song, Li Sun, Shu-Ling Zhang, Le-Tian Huang, Wei Jing, Jian-Zhu Zhao, Cheng-Bo Han

**Affiliations:** Department of Oncology, Shengjing Hospital of China Medical University, Shenyang 110004, China

## Abstract

**Purpose:**

A meta-analysis of randomized controlled trials (RCTs) was conducted to compare the difference in efficacy and safety between epidermal growth factor receptor-tyrosine kinase inhibitors (EGFR-TKIs) with antiangiogenic inhibitors (A + T) and EGFR-TKI monotherapy in patients with treatment-naïve advanced EGFR-mutant non-small-cell lung cancer (NSCLC).

**Methods:**

PubMed, Embase, Web of Science, and Cochrane electronic databases were searched for relevant RCTs. Meeting abstracts were also reviewed to identify appropriate studies. The endpoints included progression-free survival (PFS), overall survival (OS), 1- and 2-year OS rates, objective response rate (ORR), and grade ≥ 3 adverse events. All pooled outcomes were expressed using hazard ratios (HRs) or relative risk ratios (RRs).

**Results:**

Data were collected from six eligible RCTs, which included 1,244 participants (619 in the A + T group and 625 in the TKI alone group). PFS was significantly improved with A + T compared to TKI alone (HR = 0.60; *P* < 0.01) regardless of EGFR mutation types (exon 19 deletion or L858R) and brain metastasis status (with or without brain metastases). There was no significant difference in median OS between the A + T and TKI alone groups (HR = 0.933; *P* = 0.551) regardless of EGFR mutation type. The ORR for A + T combination therapy was significantly increased compared to TKI monotherapy in exon 19 deletion subgroups (RR = 0.774; *P* = 0.008). There was no difference in the positive rates of acquired T790M mutation between the two groups (RR = 0.967; *P* = 0.846). More patients in the TKI alone group received a variety of subsequent systemic treatments than those in the A + T group (RR = 0.881; *P* = 0.002).

**Conclusion:**

Addition of antiangiogenic inhibitors to first-line EGFR-TKI therapy significantly reduced the risk of disease progression for patients with advanced EGFR-mutant NSCLC regardless of EGFR mutation type and brain metastasis status. The lack of OS benefit may be explained by differences in subsequent treatments rather than drug resistance mechanisms.

## 1. Introduction

Lung cancer is the most common malignant tumor worldwide and the leading cause of cancer-related death. Non-small-cell lung cancer (NSCLC) accounts for about 85% of lung cancer cases [1]. At the time of initial diagnosis, about one-third of NSCLC patients cannot be treated with radical surgery or radiotherapy due to metastatic disease. Systemic therapies, including chemotherapy, targeted therapy, and immunotherapy, are the main treatment methods. Epidermal growth factor receptor- (EGFR-) mutant lung cancer accounts for about 10–30% of advanced NSCLCs and 40–55% of Asian lung adenocarcinoma cases [2]. The most common EGFR mutation types are exon 19 deletion and exon 21 L858R [3], which are also known as classical or sensitive mutations. Early clinical studies have confirmed first-generation EGFR tyrosine kinase inhibitors (EGFR-TKIs) as the standard for first-line treatment of advanced NSCLC patients with EGFR-sensitive mutations, with the progression-free survival (PFS) ranging from 8 to 13.8 months. Subsequently, second- and third-generation EGFR-TKIs have also been approved as the first-line treatment, with PFS of 11–18.9 months [4–9]. However, patients who initially respond to EGFR-TKIs will inevitably develop disease progression due to secondary drug resistance. In particular, patients with L858R mutations often develop resistance earlier than patients with exon 19 deletion, suggesting that they are two different types of lung cancer. In addition, some patients may experience primary drug resistance, which may be associated with concurrent mutations or tumor heterogeneity.

*In vitro* experiments have shown that blocking antiangiogenic pathways, such as vascular endothelial growth factor (VEGF) or its receptor (VEGFR), has synergistic antitumor activity and can delay the occurrence of EGFR-TKI resistance [10, 11]. VEGF/VEGFR and EGFR are two parallel but downstream cross-signaling pathways. EGFR activation upregulates the expression of VEGF/VEGFR and promotes VEGFR-mediated angiogenesis. EGFR inhibitors can reduce the expression of VEGF while inhibiting the autocrine signal of EGFR. At present, a series of prospective randomized controlled clinical trials (RCTs) have been conducted to explore the treatment strategy of EGFR-TKI combined with antiangiogenic inhibitor (A + T) to delay or overcome the occurrence of drug resistance, thereby avoiding early progression observed with EGFR-TKI treatment and improving survival benefits. Among them, EGFR-TKIs combined with bevacizumab (a monoclonal antibody against VEGF recombinant human immunoglobulin G1) or ramucirumab (a VEGFR2 monoclonal antibody that specifically binds to VEGFR2 and prevents it from binding to ligands VEGF-A, VEGF-C, and VEGF-D) showed significantly longer PFS than EGFR-TKIs alone [12–15]. However, several RCTs have reported that compared to TKI alone, A + T does not achieve overall survival (OS) benefits due to currently unknown reasons. The present meta-analysis was conducted to compare the difference in efficacy and safety between A + T and EGFR-TKIs alone for the treatment of NSCLC patients with EGFR-sensitive mutations. The A + T benefit differences in different subgroups were also analyzed to explore the advantageous groups that benefit from the A + T model.

## 2. Materials and Methods

This meta-analysis was conducted in accordance with the Preferred Reporting Items for Systematic Review and Meta-analyses (PRISMA) guidelines [16].

### 2.1. Search Strategy

PubMed, Embase, Web of Science, and Cochrane electronic databases were searched using the following terms: (“epidermal growth factor receptor” OR “EGFR”) AND (“tyrosine kinase inhibitor” OR “TKI”) AND (“anti-angiogenic inhibitor” OR “VEGF” OR “VEGFR”) AND (“lung cancer” OR “NSCLC”) to find relevant trials. Abstracts from the American Society of Clinical Oncology (ASCO), European Society of Medical Oncology (ESMO), and International Association of Lung Cancer websites were also reviewed. All reference lists for eligible studies were manually checked to ensure all relevant literature was retrieved. The search end date was June 20, 2020.

### 2.2. Inclusion Criteria

The inclusion criteria were formulated with the Participants, Intervention, Control, Outcomes, and Study designs (PICOS) strategy [17]. The inclusion criteria for literature retrieval were as follows: (1) untreated patients with advanced EGFR-mutant NSCLC; (2) therapy comparing EGFR-TKIs plus anti-angiogenic inhibitors (A + T) with EGFR-TKIs alone; (3) outcomes including at least one of the following endpoints: PFS, OS, objective response rate (ORR), and adverse effects (AEs); and (4) prospective RCT design.

### 2.3. Data Extraction and Quality Assessments

Two authors extracted data from each retrieved article or abstract and independently assessed study quality. The extractable data included authors, years of publication, patient baseline characteristics, histopathologic types and clinical stages, smoking history, EGFR mutation types, treatment regimens of study and control groups, study phase and design, survival outcome data, therapeutic response, subsequent systematic treatment, gene detection information (e.g., T790M mutation) after resistance to EGFR-TKIs, treatment duration, and AEs. The risk of bias was assessed for each RCT according to the Cochrane Collaboration guidelines [18], which comprised selection, performance, detection, attrition, and reporting bias, among others. Each item was qualified as high, low, or unclear risk of bias.

### 2.4. Statistical Analysis

Statistical analyses were performed with Stata 12 (StataCorp, College Station, Texas, USA). The primary endpoints were PFS and OS, while the secondary endpoints were 1- and 2-year OS rates, ORR, and grade ≥ 3 AEs. All pooled outcomes were expressed via hazard ratios (HRs) or relative risk ratios (RRs). OS was evaluated from the time of random assignment until death due to any cause. PFS was defined as the time from randomization to disease progression or death from any cause. PFS2 was defined as the time from randomization to second disease progression or death from any cause. HRs or RRs for outcomes of median PFS and median OS; rates for 1-, 2-, and 5-year OS; ORR; and rates of grade ≥ 3 AEs were pooled. Subgroup analyses were performed for gender, clinical stages, Eastern Cooperative Oncology Group (ECOG) performance status (PS) scores, and EGFR status with or without brain metastasis. The heterogeneity was evaluated using *I*^2^ statistics and publication bias assessed using funnel plots and Egger's test. Sensitivity analysis was performed for the primary outcome based on the leave-one-out approach.

## 3. Results

### 3.1. Literature Search and Study Characteristics

**Figure 1:**
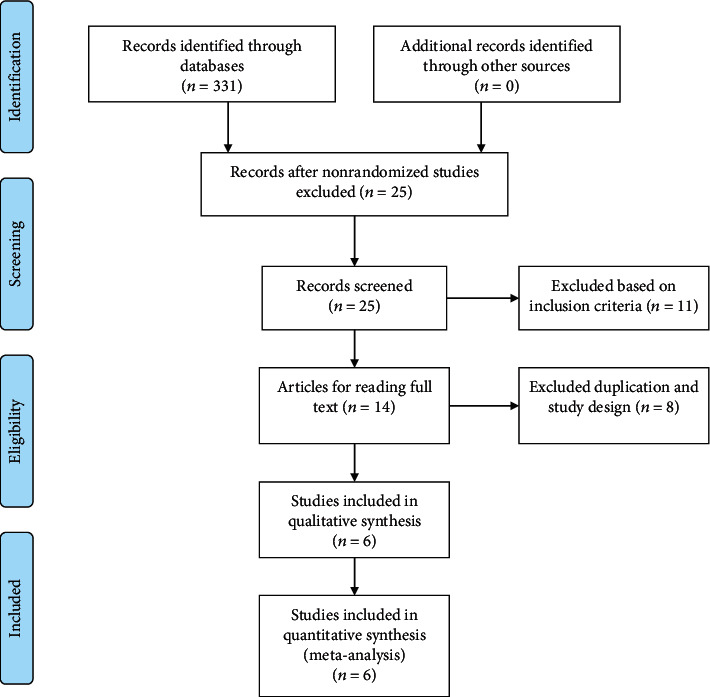
Study selection flow diagram.

A PRISMA flow diagram of the literature search process is shown in Figure 1. A total of six RCTs [12–15, 19, 20] involving 1,244 patients with EGFR-mutant advanced NSCLC who received first-line A + T combination therapy or EGFR-TKI monotherapy were included in this meta-analysis. Of them, three were phase II RCTs and three were phase III RCTs. Five RCTs compared bevacizumab plus EGFR-TKI and EGFR-TKI alone, and only one RCT compared ramucirumab plus EGFR-TKI and EGFR-TKI alone. Five RCTs used EGFR-TKI erlotinib, and only one RCT used gefitinib. The basic study characteristics are summarized in Table 1. In total, 619 patients were treated with A + T and 625 patients were treated with EGFR-TKIs alone. Furthermore, 664 patients had EGFR exon 19 deletion and 573 patients had L858R mutation. Patients with brain metastases were allowed to participate in three RCTs, of which 188 patients had brain metastases (Table 1).

### 3.2. Survival Outcomes

#### 3.2.1. PFS

**Figure 2:**
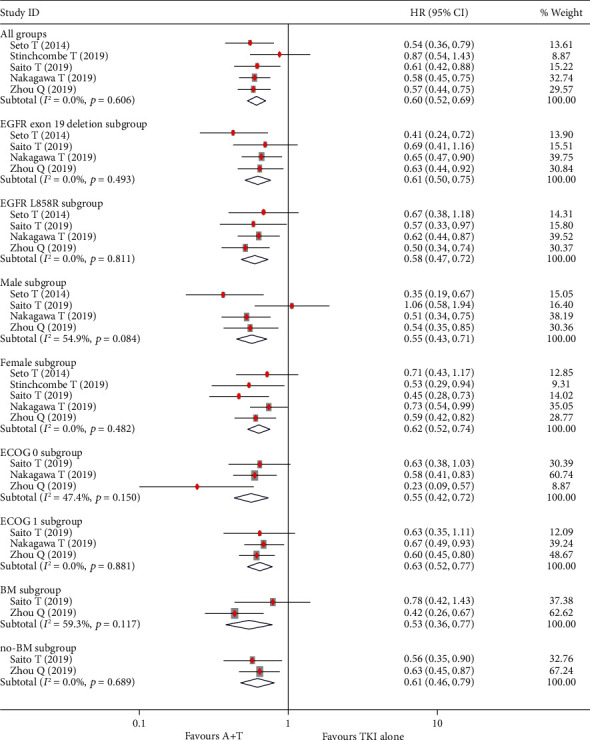
Effect of treatment on median progression-free survival (PFS) in all groups and subgroups. HR: hazard ratios; 95% CI: 95% confidence intervals.

The pooled investigator-assessed median PFS values in six RCTs were 17.9 months (95% CI, 16.3 to 19.5) and 11.9 months (95% CI, 11.1 to 12.7) in the A + T and TKI alone groups, respectively, with an HR value of 0.60 (95% CI, 0.52 to 0.69; *P* < 0.01; Figure 2). The independently reviewed pooled median PFS values in two RCTs were 17.3 months (95% CI, 15.3 to 19.2) and 11.2 months (95% CI, 10.0 to 12.4), respectively, with an HR of 0.61 (95% CI, 0.51 to 0.74; *P* < 0.01). In both exon 19 deletion and L858R mutation subgroups, patients treated with A + T combination therapy showed a significantly longer PFS than those treated with TKI monotherapy (all *P* < 0.05), with a median PFS of 18.4 months (95% CI, 16.5 to 20.3) vs. 12.2 months (95% CI, 11.0 to 13.4), respectively, in the exon 19 deletion subgroup (HR, 0.61; 95% CI, 0.55 to 0.68; *P* < 0.01) and 18.0 months (95% CI, 15.5 to 20.4) vs. 10.8 months (95% CI, 9.4 to 12.1), respectively, in the L858R subgroup (HR, 0.58; 95% CI, 0.47 to 0.72; *P* < 0.01; Figure 2). Patients with or without brain metastases were able to experience PFS benefits from A + T, with HR for brain metastases subgroup of 0.53 (95% CI, 0.36 to 0.77) and 0.61 (95% CI, 0.46 to 0.80; all *P* < 0.05; Figure 2) for no brain metastasis subgroup. In addition, patients benefited from A + T combination therapy regardless of their ECOG score (0, 1), sex (male, female), stage (IIIB, IV or relapse), or smoking status (Figure 2, Table 2).

#### 3.2.2. OS

**Figure 3:**
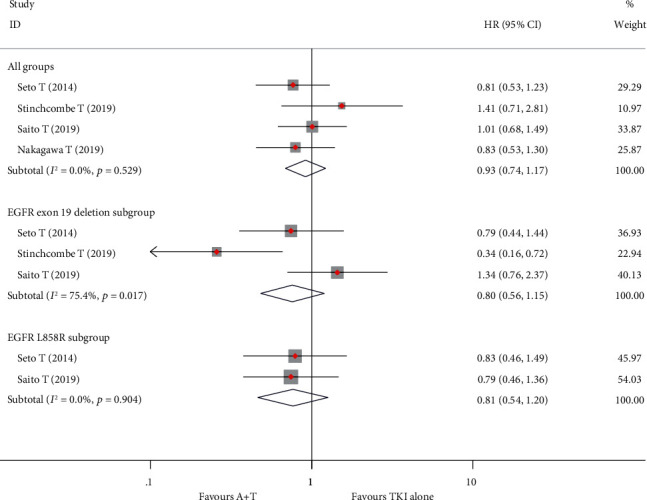
Effect of treatment on median overall survival in all groups and subgroups. HR: hazard ratios; 95% CI: 95% confidence intervals.

Three studies reported median OS but lacked 95% CIs. Thus, the pooled median OS was calculated using a weighted average of single study medians. The pooled median OS in the A + T and TKI alone groups were 46.1 and 47.4 months, respectively. Four studies recorded HRs for OS. The pooled HR using the fixed effects model was 0.933 (95% CI, 0.74 to 1.17; *P* = 0.551; Figure 3(a)). Three studies reported values of median OS and HR in exon 19 deletion and L858R subgroups. The pooled median OS for A + T and TKI alone was 46.61 months vs. not calculated in the exon 19 deletion subgroup (HR, 0.74; 95% CI, 0.35 to 1.54; *P* = 0.416) and 48.0 vs. 39.7 months in the L858R subgroup (HR, 0.81; 95% CI 0.54 to 1.20; *P* = 0.295), respectively (Figures 3(b) and 3(c)). No studies reported survival data for patients with brain metastases (Table 2).

#### 3.2.3. OS Rate and ORR

Two RCTs reported 1-year and 2-year OS rates. The combined RRs were 1.07 (95% CI, 0.61 to 1.88; *P* = 0.818) and 0.88 (95% CI, 0.64 to 1.20; *P* = 0.404), respectively. There was no significant difference in ORR between the A + T and TKI alone groups for all patients. However, in the exon 19 deletion subgroup, A + T combination therapy significantly improved ORR (RR, 0.77; 95% CI, 0.64 to 0.94; *P* = 0.008). The detailed results are summarized in Table 2.

### 3.3. T790M Proportion

Three RCTs reported the detection results for the T790M mutation in the A + T and TKI alone groups. Only one test method was used in each study. The detailed detection method, sample type, and T790M detection rate are shown in Table 3. There was no difference in the positive rates of T790M mutation between the two groups, which were 30.4% (95% CI, 21.7% to 39.1%) in the A + T group and 34.6% (95% CI, 27.3% to 42%) in the TKI alone group (RR, 0.97; 95% CI, 0.69 to 1.36; *P* = 0.846).

### 3.4. Subsequent Systemic Treatments

Four RCTs reported subsequent systemic treatments, including chemotherapy, osimertinib, other EGFR inhibitors, and immunotherapy (Table 4). There were more patients in the TKI alone group (75.3%; 95% CI, 71.4 to 79.1%) who received a variety of subsequent systemic treatments than in the A + T group (67.9%; 95% CI, 63.8 to 72%) with an RR value of 0.88 (95% CI, 0.81 to 0.96; *P* = 0.004). There was no difference in the proportion of patients receiving subsequent osimertinib treatments between the two groups, with 11.7% (95% CI, 8.5 to 14.9%) in the A + T group and 19% (95% CI, 15.1 to 22.9%) in the TKI alone group (RR, 0.76; 95% CI, 0.56 to 1.03; *P* = 0.077). A total of 19.1% and 25.4% patients received chemotherapy as a subsequent treatment in the A + T and TKI alone groups, respectively (RR, 0.84; *P* > 0.05).

### 3.5. Grade ≥ 3 Treatment-Related AEs

Grade 3 or higher AEs more commonly occurring in the A + T group than in the TKI alone group were hypertension (26.5% vs. 2.6%; RR, 1.8; 95% CI, 1.62 to 1.99), proteinuria (4.8% vs. 0.9%; RR, 1.94; 95% CI, 1.75 to 2.16), and diarrhea (3.5% vs. 1.6%; RR 1.48; 95 CI, 1.23 to 1.79; *P* for all <0.05). There was no significant difference in the incidence of rash and dermatitis, fatigue, anemia, bleeding and thrombosis, abnormal liver function, and interstitial lung disease between the two groups (Table 5).

### 3.6. Drug Treatment Duration

A total of four studies reported on the duration of treatment, three of which were bevacizumab combined with erlotinib and one was ramucirumab combined with erlotinib. The pooled results showed that the median treatment duration for bevacizumab and erlotinib was 328.0 days (95% CI, 196.6 to 459.5), 370.5 days (95% CI, 138.3 to 602.7) for the combined group, and 333.2 days (95% CI, 106.9 to 559.4) for the control group. The median treatment time for ramucirumab and erlotinib was 308 days (95% CI, 148.5 to 467.6), 395 days (95% CI, 202 to 588) for the combined group, and 314 days (144.5 to 483.5) for the control group.

### 3.7. Sensitivity Analysis

**Figure 4:**
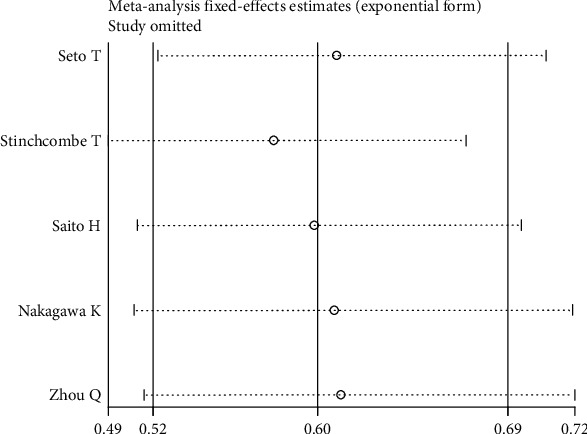
Sensitivity analysis.

Sensitivity analysis results for HRs of median PFS, in which the studies were omitted one-by-one, are summarized in Figure 4. The HR value for each study was similar to the pooled HR value and 95% CI (0.60, 0.52 to 0.69), except for Stinchcombe et al.'s study [18], which made the HR value slight lower when it was omitted (0.57, 95% CI, 0.49 to 0.67; Figure 4).

### 3.8. Quality Assessment and Publication Bias

**Figure 5:**
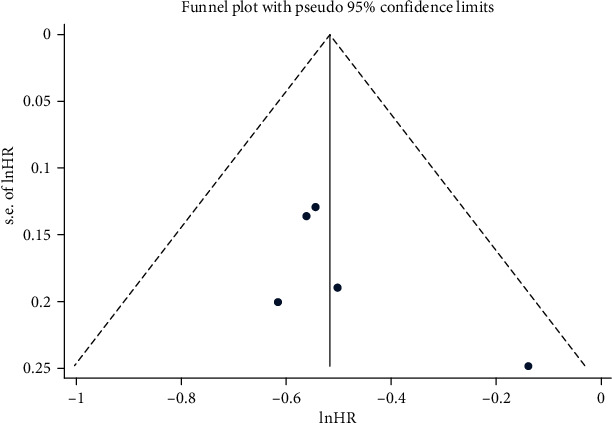
Publication bias assessment.

Cochrane risk of bias assessment of the included studies indicated that they were of high quality (Table 6), but two studies were downgraded to medium quality if they were re-evaluated according to the Grading of Recommendations Assessment, Development, and Evaluation (GRADE) approach by using the GRADEpro web application (http://gdt.gradepro.org) (Supplementary Table S1). Potential publication bias was assessed using funnel plots with PFS as the outcome. The funnel plots were symmetrical for each of the treatment groups, indicating no publication bias (*P* > 0.05; Figure 5).

## 4. Discussion

The present meta-analysis showed that the combination of antiangiogenic inhibitors and EGFR-TKIs significantly prolonged PFS in patients with advanced EGFR-mutant NSCLC compared to EGFR-TKIs alone, regardless of EGFR mutation type, brain metastasis status, or ECOG PS score. ORR in the exon 19 deletion subgroup was also improved. However, based on the current data, the combination therapy did not improve OS and led to increased but manageable toxicity. The present meta-analysis concluded that compared to EGFR-TKI monotherapy, A + T combination therapy can significantly delay the onset of drug resistance but cannot improve the OS of any subgroup of patients. The lack of OS benefit may be explained by differences in subsequent treatments rather than drug resistance mechanisms.

Two RCTs from Japan, JO25567 and NEJ026, first explored the benefits of A + T compared to TKIs alone. Their results showed that A + T significantly improved PFS [12, 13]. The PFS benefits were confirmed again by a Chinese multicenter phase III RCT CTONG1509 reported at the ESMO meeting in 2019 and by a global multicenter phase III RCT RELAY reported at the ASCO meeting in 2019 [14, 15]. Unfortunately, the OS data released by the JO25567 and NEJ026 studies showed that prolonged PFS did not ultimately translate into OS benefits. The pooled OS data from three RCTs also showed no OS differences in the overall group (median, 46.07 vs. 47.44; HR, 0.99) and the predefined subgroup of EGFR mutation types (exon 19 deletions vs. L858R). The reason may be related to the different follow-up treatment and drug resistance mechanisms.

For the follow-up treatment, pooled data from four RCTs showed that more patients in the TKI alone group received a variety of subsequent systemic treatments compared to the A + T group (75.3% vs. 65.9%; RR, 0.88; *P* = 0.004). In the NEJ026 study, 76% of patients in the A + T group and 83% in the TKI alone group received subsequent treatments, including chemotherapy (39.4% vs. 52%), osimertinib (25.9% vs. 25%), other EGFR inhibitors (8% vs. 7%), and immunotherapy (2.7% vs. 0%). In this study, the median PFS2 defined by median survival time from enrollment to progressive disease of second-line treatment was 28.6 months in the A + T group and 24.3 months in the TKI alone group (HR, 0.80; 95% CI, 0.59 to 1.10), which indicated that the additional effect of bevacizumab on erlotinib monotherapy in NSCLC with EGFR mutations gradually decreased [13]. In the RELAY study, 53.6% of patients in the A + T group received a first subsequent treatment compared to 69.3% in the TKI alone group, of which chemotherapy was 22.5% vs. 25.6%, EGFR-TKI was 74.2% vs. 72.4% (including erlotinib 50.8% vs. 35.3%; afatinib 0.8% vs. 7.8%; and osimertinib 15% vs. 22.4%), and immunotherapy was 3.3% vs. 1.9%, respectively. The proportion of patients receiving a second subsequent treatment was 28.1% in the A + T group and 33.8% in the TKI alone group, of which chemotherapy was 42.9% vs. 56.6% and osimertinib was 41.3% vs 25%, respectively [15]. In the JO25567 study, 85% and 84% of patients in the A + T and TKI alone groups, respectively, received second-line treatment, including chemotherapy (40.6% vs. 30.8%) and first-generation EGFR-TKIs (36.8% vs. 36.9%). Although there was no difference in median OS (47 vs. 47.4 months), the 5-year OS rate after long-term follow-up in the A + T group was higher than that in the TKI alone group (41% vs. 35%, respectively) [12]. In summary, compared to the A + T group, more patients in the TKI alone group received a variety of follow-up systemic treatments. These unbalanced subsequent treatments might result in similar OS in the two groups. Of note, these studies did not report any information about local treatment after progression, which is very important, because previous studies have suggested that for patients with oligoprogression or oligometastasis diseases during or after EGFR-TKI treatment, the addition of local treatment may prolong the duration of TKI treatment and even improve OS (median, 37 to 43 months) [21–23].

Furthermore, whether there are differences in drug resistance patterns between the A + T combination treatment and TKI monotherapy is worthy of discussion, because different resistant mechanisms will affect the follow-up treatments and survival. The acquired T790M mutation is the main mechanism of secondary drug resistance in patients who progressed on first- and second-generation EGFR-TKIs. Patients with T790M can benefit from subsequent third-generation EGFR-TKI osimertinib, where T790M also serves as an important prognostic factor. Previous meta-analysis showed that an acquired T790M mutation is associated with longer PFS and post-progression survival [24]. The present pooled data showed that there was no difference in the positive rate of T790M mutation between the A + T and TKI alone groups (30.4% vs. 34.6%; RR, 0.97; *P* = 0.846). In the NEJ026 study, results of the polymerase chain reaction clamp-based detection test in tissue/blood after drug resistance in the two groups showed that the proportion of T790M was 24% vs. 26%, respectively. The proportion of patients receiving follow-up osimertinib in the two groups was similar [13, 25]. In the RELAY study after drug resistance, liquid biopsy samples were detected using a next-generation sequencing (NGS) method. The positive rate of T790M mutation was 43% in the A + T group and 47% in the erlotinib alone group [15]. In Stinchcombe et al.'s study, although T790M proportion was not reported by the group, cell-free DNA test results for 36 postprogression patients showed that T790M was detected in five (42%) of 12 patients with EGFR-sensitive mutations [20]. Similarly, the positive rate of T790M mutation obtained via NGS-based testing in tissue/blood samples in the CTONG1509 study was 33% in the combination group and 44% in the erlotinib alone group, but the difference was not statistically significant [14]. A real-world study also compared first-line A + T combination therapy (60 cases) to TKI monotherapy (120 cases) in patients with advanced EGFR-sensitive mutant NSCLC [26]. Tumor biopsy and NGS testing after progression were performed again in the two groups. It was found that the positive rate of T790M in the A + T group (36%) was significantly lower than that in the TKI group (52%). However, in the RELAY + study presented at the 2020 ASCO meeting, seven (78%) of nine patients in the ramucirumab plus gefitinib group who experienced acquired drug resistance had a T790M mutation [27]. In summary, there was no consistent evidence showing that the A + T mode can change the EGFR-TKI resistance mechanism. Furthermore, it is unclear whether there were differences in resistance mechanisms other than T790M.

In addition, it was unclear whether ethnic differences affected survival benefit. The effect of race on OS was shown in the FLAURA study, which indicated that osimertinib improved OS in the non-Asian subgroup, but not in the Asian subgroup. In Stinchcombe et al.'s study [20], there was no difference in median OS between the A + T and TKI alone groups (32.4 vs. 50.6 months), but the value of OS in the A + T group was obviously lower than that in the TKI alone group. Unlike the two Japanese studies [12, 13], this study enrolled only a small number of cases (*n* = 88), and 85% of the patients were white.

Although OS was not improved significantly in a series of A + T studies, PFS benefits were observed regardless of exon 19 deletion or L858R mutation subgroups. The present meta-analysis indicated that compared to EGFR-TKI monotherapy, A + T combination therapy significantly prolonged median PFS by 6.18 and 7.22 months and decreased the risk of disease progression by 39% and 42% in the exon 19 deletion and L858R subgroups, respectively. Many clinical studies have shown that sensitivity and survival of patients with L858R are worse than those with exon 19 deletion. The reason may be that the tyrosine kinase domain of the L858R mutant has a weaker binding force to EGFR-TKIs than that of the exon 19 deletion [28] and co-mutation is more frequent in the L858R-mutant NSCLC [29–31]. This combination seems to be more effective for L858R-mutant NSCLC. Basic experiments have shown that L858R mutant lung cancer cells have more invasive molecular and pathological characteristics that are closely related to angiogenesis [32]. Theoretically, dual blocking of the VEGF/EGFR pathway can improve the antitumor effect for these relatively aggressive L858R-mutant populations.

Although A + T has a significant PFS improvement in L858R patients compared to TKI alone, it also does not translate into OS benefits. However, the OS values in both A + T and TKI alone groups (46.1 vs. 47.4 months) are better than their historical values (range, 20 to 38.6 months) [6, 7, 9]. This may be related to a greater proportion of patients receiving more follow-up treatments, in which the “A+” mode (including A + T regimen in the combination group and A + chemotherapy regimen after resistance to TKI in the control group) may play an important role. The BEYOND study showed that OS was increased by 6.6 months (24.3 vs. 17.7 months) and ORR was increased by 28% (54% vs. 26%) in a group of unselected lung adenocarcinoma patients (including some EGFR-sensitive mutation-positive patients) [33]. It suggested that the “A+” mode is of great survival benefit for patients with EGFR mutations. Although whether A + T followed by chemotherapy or TKI followed by A + chemotherapy is a suitable treatment mode for patients with exon 19 deletion or L858R needs more research exploration; importantly, using the “A+” mode of antiangiogenic therapy is of great significance for these patients.

The benefits of specific clinical features, such as brain metastasis status and PS score, are also worthy of discussion. It has been reported that patients with EGFR-mutant lung adenocarcinoma are more likely to develop brain metastases (39.2% vs. 28.2%; *P* = 0.038) and meningeal metastases (9.4% vs. 1.7%; *P* < 0.01) than wild-type patients [34, 35]. Previous preclinical and clinical studies have shown that bevacizumab-containing regimens have the ability to delay brain metastasis [36, 37]. In the NEJ026 and CTONG1509 studies, 32% and 29% of enrolled patients had brain metastases, respectively. The present pooled data for three RCTs showed that compared to TKI monotherapy, A + T combination therapy resulted in PFS benefit for patients with brain metastases. The HR value in the brain metastasis subgroup was 0.53, which was lower than 0.60 in the nonbrain metastasis subgroup, although PFS time was only extended by 1.5 months (12.7 vs. 11.2 months). Although there were no patients with brain metastases enrolled in the RELAY study, fewer patients in the A + T group developed brain metastases than TKI alone group (two vs. eight cases). In addition, a retrospective cohort study [38] showed that compared to EGFR-TKIs alone, A + T treatment significantly increased intracranial ORR (66.1% vs. 41.6%; *P* = 0.001) and systemic ORR (74.6% vs. 57.1%; *P* = 0.019) and prolonged intracranial PFS (14 vs. 8.2 months; *P* < 0.01), system PFS (14.4 vs. 9 months; *P* < 0.01), and OS (29.6 vs. 21.7 months; *P* < 0.01). Whether the current A + T mode is comparable to the third-generation osimertinib in terms of antitumor activity in the central nervous system is a question that still needs to be answered. In the FLAURA study, the PFS time for osimertinib patients with brain metastases was 15.2 months, which was 5.6 months higher than that for erlotinib/gefitinib. The risk of disease progression decreased by 53% (HR, 0.47; 95% CI, 0.30 to 0.74). In summary, both A + T and osimertinib can reduce the risk of brain metastases progression compared to TKI monotherapy, but the improvement degree of osimertinib has more clinical significance. A pooled analysis of the patient PS scores was also performed. In ECOG PS score 0 and 1 subgroups, the risk of disease progression for the combination therapy compared to monotherapy was decreased by 45% and 35%, respectively, indicating that both PS score 0 and 1 can benefit from combination therapy. In these RCTs, patients with poor PS scores (≥2) were often excluded from clinical trials. Therefore, whether patients with PS ≥ 2 can benefit from A + T combination therapy remains unclear.

The difference between A + T and second- or third-generation EGFR-TKIs as the first-line treatment is an interesting topic, and there is no direct comparison with large samples yet. It may be a good strategy to make choices based on the different patient characteristics. First, in the exon 19 deletion subgroup, pooled results of four studies showed that the median PFS in the A + T group was 18.39 months (HR, 0.61, 95% CI, 0.55 to 0.68). Although it did not exceed the PFS of 21.4 months for osimertinib monotherapy in the FLAURA study (HR, 0.43; 95% CI, 0.32 to 0.56), this is already a very similar value. Special attention should be paid to the L858R mutation subgroup. The pooled median PFS in the A + T group was 17.98 months (HR, 0.58; 95% CI, 0.47 to 0.72). In the L858R subgroups of the RELAY and CTONG1509 studies, the median PFS for the A + T group was 19.4 months (HR, 0.62; 95% CI, 0.44 to 0.87) and 19.5 months (HR, 0.51; 95% CI, 0.33 to 0.79), respectively [14, 15]. The ARCHER1050 study showed that the second-generation EGFR-TKI dacomitinib significantly prolonged PFS (12.3 vs. 9.8 months; HR, 0.63) and OS in the L858R subgroup compared to gefitinib [8]. In the FLAURA study, the median osimertinib PFS in the L858R subgroup was 14.4 months (HR, 0.51; 95% CI, 0.36 to 0.71) and there was no OS benefit compared to first-generation EGFR-TKIs (HR, 1.0; 95% CI, 0.71 to 1.41) [9]. In summary, the A + T combination therapy could improve PFS in L858R mutant patients better than second-generation dacomitinib and third-generation osimertinib monotherapy. Moreover, these patients still had the opportunity to benefit from third-generation TKI treatment after progression to the A + T treatment.

Results of the meta-analysis showed that treatment-related toxicity in the A + T group was significantly higher than that in the TKI alone group. The main grade 3 or higher AEs in the A + T group were hypertension, proteinuria, acne-like rash, diarrhea, fatigue, and anemia. There was no significant difference in the incidence of bleeding and thrombosis, abnormal liver function, and interstitial lung disease between the two groups. In the JO25567 study, 45.3% of patients in the A + T group discontinued treatment due to AEs associated with bevacizumab, but continued to take erlotinib. There was no difference in the proportion of erlotinib discontinuation rates between the two groups, which were 17.3% and 18.2%, respectively [12]. In the NEJ026 and Stinchcombe et al. studies, 29% and 26% of patients in the A + T group discontinued treatment due to AEs associated with bevacizumab [13]. In the RELAY study, the proportion of discontinuation in the A + T group due to serious AEs was lower, mainly due to elevated transaminase, paronychia, acne-like rash, and proteinuria, with the proportion rates ranging from 0.8% to 1.3% [15]. According to the present meta-analysis, the incidence of hypertension, proteinuria, and rash was the highest, causing the highest proportion of drug discontinuation or dose adjustment. However, the median treatment duration of antiangiogenic targeted drugs and TKI drugs in the combination treatment group was not inferior to the TKI treatment duration in the TKI alone group, suggesting that toxicity of the A + T combination therapy was increased, although most of it was controllable. Antiangiogenic drug toxicity did not reduce the treatment duration of the TKI drugs.

There were some limitations in this study. Significant heterogeneity was observed in the included studies. First, the sample size of each study group was different, and two different antiangiogenic drugs and two EGFR-TKIs were used in these studies. Second, patient clinical characteristics in each study group were inconsistent. For example, two studies included a certain proportion of patients with brain metastases, which may have an impact on the summarized efficacy and survival results.

## 5. Conclusion

Meta-analysis based on RCT data showed that compared to EGFR-TKIs alone, antiangiogenic inhibitors (anti-VEGF/VEGFR monoclonal antibody) combined with EGFR-TKIs, i.e., A + T treatment mode, significantly improved PFS in patients with EGFR-sensitive mutations regardless of brain metastasis status, EGFR mutation types (exon 19 deletion and L858R), and PS scores (0 and 1). However, prolonged PFS did not yield the OS benefits in overall groups or any subgroup. This may be explained by different subsequent treatments rather than drug resistance mechanisms. There was no consistent evidence showing that the A + T mode changes the EGFR-TKI resistance mechanism. The combination therapy significantly increased AEs, but the toxicity was controllable. Importantly, EGFR-TKI treatment duration was not reduced by antiangiogenic drug-induced discontinuation. In view of the worse PFS for EGFR-TKI monotherapy in patients with L858R mutation and the best PFS benefit observed in the A + T mode, first-line A + T combination therapy for L858R-mutant patients is recommended in the absence of contraindications for antiangiogenic therapy in order to delay disease progression. Further analyses of drug resistance mechanisms and follow-up treatments are needed. Ongoing RCTs ECOG ACRIN (NCT04181060) and HCRN (NCT03909334) are investigating first-line treatment strategies of osimertinib combined with bevacizumab or ramucirumab in patients with EGFR-mutant advanced or metastatic NSCLC and may produce important data in the future.

## Figures and Tables

**Table 1:** The characteristics of the included studies.

Study/author name	JO25567 [12]		Kitagawa C [19]		Stinchcombe T [20]		NEJ026 [13]		RELAY [15]		CTONG1509 [14]	
Study phase	Phase II RCT		Phase II RCT		Phase II RCT		Phase III RCT		Phase III RCT		Phase III RCT	
Study arms	Bev + Erl	Erl	Bev + Gef	Gef	Bev + Erl	Erl	Bev + Erl	Erl	Ram + Erl	Erl	Bev + Erl	Erl
No. of patients (*n*)	75	77	6	10	43	45	112	112	224	225	157	154
Median age, years	67	67	73.5	72.5	65	63	67	68	65	64	NR	NR
Male (*n*, %)	30 (40%)	26 (33.8%)	1 (16.7%)	3 (30%)	12 (27.9%)	14 (31.1%)	41 (36.6%)	39 (34.8%)	83 (37.1%)	83 (36.9%)	60 (38.2%)	58 (37.7%)
Never smoker	42 (56%)	45 (58.4%)	4 (66.7%)	8 (80%)	25 (58.1%)	23 (51.1%)	65 (58%)	64 (54.5%)	134 (59.8%)	139 (61.8%)	NR	NR
Former smoker	9 (12%)	6 (7.8%)	2 (33.3%)	2 (20%)	14 (32.6%)	15 (33.3%)	6 (5.4%)	7 (6.3%)	NR	NR	NR	NR
EGFR L858R (*n*, %)	35 (46.7%)	37 (48.1%)	2 (33.3%)	3 (30%)	14 (32.6%)	15 (33.3%)	56 (50%)	57 (50.9%)	99 (44.2%)	105 (46.7%)	75 (47.8%)	75 (48.7%)
ECOG PS 1 (*n*, %)	32 (42.7%)	36 (46.8%)	4 (66.7%)	3 (30%)	19 (44.2%)	26 (57.8%)	48 (42.9%)	42 (37.5%)	108 (48.2%)	106 (47.1%)	132 (84.1%)	137 (89%)
Adenocarcinoma (*n*, %)	74 (98.7%)	76 (98.7%)	6 (100%)	9 (90%)	NR	NR	110 (98.2%)	112 (100%)	215 (96%)	218 (96.9%)	157 (100%)	154 (100%)
Stage IIIB (*n*, %)	1 (1%)	0	0	1 (10%)	NR	NR	8 (7.1%)	8 (7.1%)	NR	NR	4 (2.6%)	6 (3.9%)
Stage IV (*n*, %)	60 (80%)	62 (80.5%)	6 (100%)	9 (90%)	39 (90.7%)	39 (86.7%)	82 (73.2%)	84 (75%)	195 (87.1%)	189 (84%)	141 (89.8%)	134 (87%)
Recurrence (*n*, %)	14 (18.6%)	15 (19.5%)	NR	NR	NR	NR	22 (19.6%)	20 (19.6%)	NR	NR	12 (7.6%)	14 (9.1%)
Brain metastases (*n*, %)	NR	NR	NR	NR	11 (25.6%)	14 (31.1%)	36 (32.1%)	36 (32.1%)	NR	NR	44 (28%)	47 (30.5%)
Treatment duration, median (days)	Bev:326 Erl:431	254	NR		NR		Bev: 350 Erl: 405	364	Bev: 308 Erl: 395	314	Bev: 462 Erl: 551	377

Bev: bevacizumab; Erl: erlotinib; Ram: ramucirumab; 19del: EGFR exon 19 deletion; L858R: EGFR L858R mutation; ECOG PS: Eastern Cooperative Oncology Group performance status scores; NR: not reported.

**Table 2:** Pooled results of survival and response rate in the A + T and TKI alone groups.

Endpoints	Study numbers	A + T vs. TKI alone	HR/RR (95% CI)	*P* value
PFS		Median (95% CI), months	HR (95% CI)	
PFS (INV) (overall)	6	17.9 (16.3–19.5) vs. 11.9 (11.1–12.8)	0.596 (0.516–0.689)	<0.01
PFS (IRC) (overall)	2	17.3 (15.3–19.2) vs. 11.2 (10.0–12.4)	0.612 (0.506–0.740)	<0.01
PFS in EGFR 19del	4	18.4 (16.5–20.3) vs. 12.21 (11.0–13.4)	0.611 (0.551–0.677)	<0.01
PFS in EGFR L858R	4	18.0 (15.5–20.4) vs. 10.76 (9.4–12.1)	0.580 (0.468–0.718)	<0.01
PFS in BM	2	12.7 (9.7–NR) vs. 11.2 (8.8–14.7)	0.529 (0.364–0.770)	0.001
PFS in no-BM	2	18 (15.4–NR) vs. 15.1 (11.1–16.1)	0.606 (0.462–0.797)	<0.01
PFS in ECOG PS 0	3	NR	0.548 (0.417–0.721)	<0.01
PFS in ECOG PS 1	3	NR	0.646 (0.507–0.786)	<0.01
PFS2 (overall)	2	NR	0.756 (0.612–0.934)	0.010
PFS in male	4	NR	0.553 (0.433-0.706)	0.003
PFS in female	5	NR	0.621 (0.519-0.742)	<0.01
PFS in stage IIIB	2	NR	0.393 (0.125-1.234)	0.110
PFS in stage IV	3	NR	0.600 (0.505-0.713)	<0.01
PFS in recurrence	3	NR	0.440 (0.239-0.811)	0.008
PFS in never-smoker	2	NR	0.645 (0.495-0.840)	0.001
OS		Median (95% CI), months	HR (95% CI)	*P* value
OS (overall)	4	46.1 vs. 47.4	0.933 (0.743–1.172)	0.551
OS in EGFR 19del	3	46.6 vs. NC	0.736 (0.352–1.539)	0.416
OS in EGFR L858R	2	48.0 vs. 39.7	0.808 (0.543–1.204)	0.295
OSR		% (95% CI)	RR (95% CI)	*P* value
1-year OSR (overall)	3	93.4% (90.6–96.2%) vs. 93.4% (90.7–96.2%)	1.068 (0.607–1.880)	0.818
2-year OSR (overall)	2	79.2% (69.9–88.5%) vs. 78.3% (73.6–82.9%)	0.876 (0.641–1.196)	0.404
ORR		% (95% CI)	RR (95% CI)	*P* value
ORR (overall)	6	77.3% (74–80.5%) vs. 74.4% (71–77.8%)	0.884 (0.769–1.106)	0.083
ORR in EGFR 19del	2	76.9% (70.7–80%) vs. 67.2% (60.2–74.1%)	0.774 (0.641–0.936)	0.008
ORR in EGFR L858R	2	67.9% (60.6–75.3%) vs. 64.9% (57.6–72.3%)	0.937 (0.759–1.104)	0.435

TKI: tyrosine kinase inhibitor; A + T: angiogenic inhibitors combined with TKI; 19del: exon 19 deletion; PFS: progression-free survival; PFS2: time from randomization to second disease progression or death from any cause; OS: overall survival; OSR: OS rate; ORR: objective response rate; DCR: disease control rate; NR; not reported; NC: not calculated; ECOG: Eastern Cooperative Oncology Group; PS: performance status; BM; INV: investigator-assessed; IRC: independent review committee-assessed; HR: Hazard ratios; RR: Relative risk ratios; 95% CI: 95% confidence intervals.

**Table 3:** Pooled results of acquired T790M detection rates in the A + T and TKI alone groups.

Study	Treatment arm	Test samples	Test methods	Test cases (*n*)	Cases of T790M (*n*)	T790M rate	Pooled T790M rate	RR/P value (A + T vs. TKI)
NEJ026 [13, 25]	Bev + Erl	Plasma	(PNA-LNA) PCR clamp	42	8	19%	A + T 30.4% (95% CI: 21.7–39.1%) vs. TKI 34.6% (95% CI: 27.3–42%)	RR = 0.967 (95% CI: 0.668–1.358), *P* = 0.846
	Erl	Plasma	(PNA-LNA) PCR clamp	45	11	20.8%		
	Bev + Erl	Tissue	(PNA-LNA) PCR clamp	7	4	57.1%		
	Erl	Tissue	(PNA-LNA) PCR clamp	9	4	44.4%		
RELAY [15]	Ram + Erl	Plasma	NGS-360	44	19	43%		
	Erl	Plasma	NGS-360	75	35	47%		
CTONG1509 [14]	Bev + Erl	Tissue	NGS-448	6	2	33%		
	Erl	Tissue	NGS-448	12	5	42%		

Bev: bevacizumab; Erl: erlotinib; Ram: ramucirumab; NGS: next-generation sequencing; RR: relative risk ratios; TKI: tyrosine kinase inhibitor; A + T: angiogenic inhibitors combined with TKI; PNA-LNA PCR clamp: peptide nucleic acid locked nucleic acid polymerase chain reaction clamp.

**Table 4:** Subsequent systemic treatments in the A + T and TKI alone groups.

Study	A + T group	TKI alone group
Rates of total subsequent systemic treatments (*n*/*N*) (%)		
JO25567 [12]	64/75 (85.3%)	65/77 (84.4%)
Stinchcombe [20]	23/43 (53.5%)	21/45 (46.7%)
NEJ026 [13]	85/112 (75.9%)	93/112 (83%)
RELAY [15]	120/224 (53.6%)	156/225 (69.3%)
Pooled rates	67.9% (95% CI: 63.8–72%)	75.3% (95% CI: 71.4–79.1%)
Pooled RR	0.881 (95% CI: 0.808–0.960), *P* = 0.002	
Rates of subsequent osimertinib treatment (*n*/*N*) (%)		
Stinchcombe [20]	10/23 (43.5%)	13/21 (61.9%)
NEJ026 [13]	29/85 (34.1%)	28/93 (30.1%)
RELAY [15]	18/120 (15%)	35/156 (22.4%)
Pooled rates	22% (95% CI: 16.8–27.2%)	27.2% (95% CI: 22.1–32.4%)
Pooled RR	0.858 (95% CI: 0.642–1.146), *P* = 0.299	
Rates of subsequent chemotherapy (*n*/*N*) (%)		
JO25567 [12]	26/64 (40.6%)	20/65 (30.8%)
NEJ026 [13]	44/85 (51.8%)	57/93 (61.3%)
RELAY [15]	27/120 (22.5%)	40/156 (25.6%)
Pooled rates	34.8% (95% CI: 29.4–40.3%)	35.9% (95% CI: 30.8–40.9%)
Pooled RR	0.969 (95% CI: 0.79–1.189), *P* = 0.766	

TKI: tyrosine kinase inhibitor; A + T: angiogenic inhibitors combined with TKI.

**Table 5:** Pooled incidence of grade ≥3 treatment-related adverse events in the two groups.

Events	Study numbers	A + T group, % (95% CI)	TKI group, % (95% CI)	RR (95% CI)	*P* value
Hypertension	6	26.5 (23.1-29.9)	2.8 (1.5-4.1)	4.98 (3.54-6.99)	<0.01
Proteinuria	6	4.8 (3.1-6.4)	0.9 (0-2.6)	13.50 (4.19-43.46)	<0.01
Hemorrhage	4	2.4 (0.9-3.9)	1.5 (0.2-2.8)	1.65 (0.62-4.34)	0.313
Thrombosis	3	1.4 (0.2-2.5)	1.7 (0.4-2.9)	0.79 (0.30-2.11)	0.644
Dermatitis acneiform	3	14.9 (10.6-19.2)	9.6 (6.2-13.1)	1.51 (0.97-2.37)	0.069
Diarrhea	6	3.5 (2.1-5.0)	1.6 (0.4-2.7)	2.58 (1.35-4.91)	0.004
Paronychia	3	2.8 (1.2-4.5)	3.1 (1.4-4.7)	1.02 (0.48-2.17)	0.960
Pruritus	2	1.0 (0-2.1)	0.9 (0-2.1)	1.42 (0.28-7.13)	0.670
Rash	6	2.3 (1.4-3.7)	3.9 (2.4-5.3)-	1.12 (0.81-1.55)	0.494
Stomatitis	3	1.3 (0.3-2.5)	1.3 (0.2-2.3)	1.02 (0.33-3.14)	0.972
Dry skin	3	0.6 (0-1.5)	2.2 (0.3-4.1)	0.65 (0.17-2.46)	0.526
Fatigue	3	1.5 (0.2-2.8)	0.5 (0-1.3)	3.09 (0.75-12.75)	0.119
Decreased appetite	4	2.0 (0.7-3.4)	1.3 (0.3-2.4)	1.53 (0.55-4.25)	0.416
Anemia	4	1.8 (0.6-3.0)	0.6 (0-1.4)	2.33 (0.78-6.95)	0.129
Bilirubin	3	1.4 (0-2.9)	1.0 (0-2.0)	0.78 (0.19-3.11)	0.725
AST	3	4.4 (2.3-6.4)	5.2 (3-7.4)	0.96 (0.558-1.66)	0.893
ALT	3	3.0 (1.3-4.7)	4.1 (2.1-6.1)	0.83 (0.42-1.64)	0.589
Interstitial lung disease	2	0.9 (0-2.1)	0.9 (0-2.1)	0.51 (0.05-5.57)	0.580

ALT: alanine aminotransferase; AST: aspartate aminotransferase; TKI: tyrosine kinase inhibitor; A + T: angiogenic inhibitors combined with TKI.

**Table 6:** Evaluation of the quality of randomized controlled trials.

Author (year)	Selection bias (randomization sequence generation)			Selection bias (allocation concealment)			Performance bias			Detection bias			Attrition bias			Reporting bias			Other biases		
	High risk	Low risk	Unclear	High risk	Low risk	Unclear	High risk	Low risk	Unclear	High risk	Low risk	Unclear	High risk	Low risk	Unclear	High risk	Low risk	Unclear	High risk	Low risk	Unclear
Seto (2014) [12]		√			√		√			√				√			√			√	
Kitagawa (2019) [19]		√			√				√			√		√			√			√	
Stinchcombe (2019) [20]		√			√				√			√		√			√			√	
Saito (2019) [13]		√			√		√			√				√			√			√	
Nakagawa (2019) [15]		√			√			√			√			√			√			√	
Zhou (2019) [14]		√			√				√			√		√			√			√	

## Data Availability

All data generated or analyzed during this study are included in this published article.

## Supplementary Materials

Supplementary Materials Table S1: GRADE quality evaluation of RCTs.
