# Supplementary material for: Rational Application of First-Line EGFR-TKIs Combined with Antiangiogenic Inhibitors in Advanced EGFR-Mutant Non-Small-Cell Lung Cancer: A Systematic Review and Meta-Analysis
Source: Biomed Res Int. 2021 Jan 28;2021:8850256. doi: 10.1155/2021/8850256 (PMC7861921; doi:10.1155/2021/8850256)
Supplement: Supplementary Materials — Table S1: GRADE quality evaluation of RCTs. [file 8850256.f1.docx]

| Certainty assessment | | | | | | | Summary of findings | | | | Certainty | Importance |
| --- | --- | --- | --- | --- | --- | --- | --- | --- | --- | --- | --- | --- |
| Author  (year) | Study design | Risk of bias | Inconsistency | Indirectness | Imprecision | Other considerations | events of patients | | Effect | |  |  |
|  |  |  |  |  |  |  | A+T  (n/N, %) | TKI alone (n/N, %) | RR (95% CI) | AR (n/1000) |  |  |
| Seto (2014) | Randomized trial | not serious | not serious | not serious | not serious | All plausible residual confounding would reduce the demonstrated efffect | 45/75 (60.0%) | 57/77 (74.0%) | 0.811 (0.646 to1.017) | 140 /1000 | ⨁⨁⨁⨁ HIGH | Critical |
| Kitagawa (2019) | Randomized trial | serious | not serious | not serious | serious | All plausible residual confounding would reduce the demonstrated efffect |  |  | not estimable |  | ⨁⨁⨁◯ MODERATE | Critical |
| Stinchcombe (2019) | Randomized trial | serious | not serious | not serious | serious | All plausible residual confounding would reduce the demonstrated efffect |  |  | not estimable |  | ⨁⨁⨁◯ MODERATE | critical |
| Saito (2019) | Randomized trial | not serious | not serious | not serious | not serious | All plausible residual confounding would reduce the demonstrated efffect | 52/112 (46.4%) | 65/112  (58%) | 0.80 (0.621 to 1.031) | 116/1000 | ⨁⨁⨁⨁ HIGH | critical |
| Nakagawa (2019) | Randomized trial | not serious | not serious | not serious | not serious | All plausible residual confounding would reduce the demonstrated efffect | 120/216 (55.6%) | 157/221 (71.0%) | 0.782 (0.676 to0.905) | 154/1000 | ⨁⨁⨁⨁ HIGH | critical |
| Zhou (2019) | Randomized trial | not serious | not serious | not serious | not serious | publication bias strongly suspected all plausible residual confounding would reduce the demonstrated efffect | 100/157 (63.7%) | 121/154 (78.6%) | 0.811 (0.702 to 0.93) | 149/1000 | ⨁⨁⨁⨁ HIGH | critical |

Table S1: GRADE quality evaluation of RCTs
